# A randomised controlled clinical and cost effectiveness trial of an online integrated bipolar parenting intervention (IBPI) compared to treatment as usual in improving child emotional and behavioural outcomes: a study protocol

**DOI:** 10.1186/s12888-025-07214-3

**Published:** 2025-08-26

**Authors:** Steven H. Jones, Sarah Byford, Elizabeth Coleman, Cathy Creswell, Lucy Cryle, Anne Duffy, Stephanie Fortier, Catherine E. Hewitt, Fiona Lobban, Christopher Lodge, Richard Morriss, Jasper Palmier-Claus, Lesley Sinclair, Christopher J. Sutton, Judith Watson, Nahel Yaziji, Eirian Kerry

**Affiliations:** 1 Spectrum Centre for Mental Health Research, Division of Health Research, Faculty of Health and Medicine, Lancaster University, Lancaster, UK; 2 Institute of Psychiatry, Psychology & Neuroscience, King’s College London, London, UK; 3 York Trials Unit, University of York, York, UK; 4 Departments of Experimental Psychology and Psychiatry, University of Oxford, Oxford, UK; 5 Department of Psychiatry, Queens University, Kingston, ON Canada; 6 Lancashire and South Cumbria NHS Foundation Trust, Lancashire, UK; 7 Faculty of Medicine and Health Sciences, University of Nottingham, Nottingham, UK; 8 Lancashire Clinical Trials Unit, University of Central Lancashire, Preston, UK

**Keywords:** Bipolar disorder, Digital intervention, Parenting intervention, Randomised controlled trial

## Abstract

**Background:**

Bipolar disorder (BD) is a severe mental health problem linked to substantial personal and social costs. Many individuals living with bipolar disorder are parents. Due to the nature of the condition, parents with BD often experience challenges in delivering consistent parenting. In addition, up to 60% of their children experience at least one mental health problem in childhood and are at increased risk of future severe mental health problems including bipolar disorder. This paper describes the rationale and protocol for a definitive randomised controlled trial of a new digital intervention (Integrated Bipolar Parenting Intervention; IBPI) to support effective parenting in the context of BD.

**Methods and design:**

The randomised controlled clinical and cost-effectiveness trial compares IBPI plus treatment as usual (TAU) with TAU alone. Parents with BD with a child aged 4–11 years old and living in the UK will be recruited through the NHS, mental health charities, and social media. Participants will be screened to confirm a clinical diagnosis of BD. They will then complete baseline assessments and be randomised to receive either IBPI + TAU or TAU with follow up assessments after 24 and 48 weeks. The primary clinical outcome is child emotional and behaviour problems measured by the Strengths and Difficulties Questionnaire at 24 weeks. The primary economic evaluation will be a cost-utility analysis at 24-weeks with quality-adjusted life years (QALYs) measured using the Child Health Utility 9 Dimensions measure of health-related quality of life. Secondary outcomes include parental mood and confidence and family functioning at 24 and 48 weeks, and child emotional and behavioural problems and health economic outcomes at 48 weeks.

**Discussion:**

Despite the challenges faced by children of parents with BD and the parents themselves, research on how to improve their lives is lacking. This will be the first definitive trial of a tailored intervention that aims to improve child and parent outcomes. Results will be reported in line with CONSORT guidance for clinical and health economic findings.

**Trial registration:**

ISRCTN Registry (ISRCTN15962574) registered on 03/05/2023.

## Introduction

### Background and rationale

Coping with instability of mood, activity and social rhythms is a key problem for individuals living with bipolar disorder (BD) [1]. BD can significantly impact a person’s life, with high rates of alcohol and substance use, suicide risk, and poor quality of life [2–7].

Children of parents with BD experience a range of challenges. The parenting they experience can be variable, as parents with BD can experience fluctuating moods that can impact basic parenting tasks [8–10]. Children of parents with BD have higher rates of anxiety and depression compared with children of parents without mental health issues [11]. In addition, parents with BD themselves report high levels of parenting stress and low levels of parenting confidence [12, 13]. Parenting programmes exist, addressing a range of child difficulties such as Attention Deficit Hyperactivity Disorder (ADHD), conduct disorder and antisocial behaviour [14], and emotional problems [15, 16]. They focus on building parental skills through providing support and information to parents based on social learning and cognitive behavioural theories [17]. However, specific parenting interventions for parents with BD are lacking.

Taking account of the elevated risk of BD and other mental health issues in the children of parents with BD, it is important to provide strategies for parents of young children, so that early intervention support is provided [18, 19].

There is good reason to think that parents with BD need interventions tailored to their specific concerns, (rather than generic approaches), including the challenges of providing consistent parenting alongside mood instability as well as the particular needs of children of parents with BD [20, 21]. Parents with BD report reluctance to acknowledge parenting challenges when speaking with mental health services due to stigma and fear of losing child custody [20, 22]. They do however report that they want tailored parenting self-management interventions [9]. For parents with BD, a parenting programme would need to provide de-stigmatising information about living with BD as well as provide parenting support linked to the specific challenges that their children experience [23, 24].

In two previous randomised controlled trials, we have shown that offering online parenting support alongside self-management support is feasible and safe for parents with BD and their children [23, 25]. This paper describes the protocol and rationale for a definitive randomised controlled clinical and cost-effectiveness trial of an online integrated bipolar parenting intervention (IBPI). The intervention was updated to incorporate learning from the previous feasibility and acceptability studies [23, 25]. Analysis of web usage data from the feasibility study of the IBPI intervention revealed low engagement levels, particularly with the parenting modules [23]. Qualitative interviews and public and patient involvement (PPI) work identified several areas requiring improvement in the new version of IBPI. Specifically, participants emphasized the need for integration of content addressing both parenting and the management of bipolar experience, as well as a clearer focus on key parental concerns. Improvements in the intervention’s design and functionality were necessary to enhance accessibility across both PC and mobile platforms. A critical change involved ensuring that all parenting-related examples were explicitly tailored to individuals with BD. In the earlier iteration, the inclusion of general parenting scenarios was perceived as less relevant and, in some cases, stigmatising.

The updated IBPI intervention has been developed in partnership with people with lived experience of BD and parenting as well as clinical experts [26, 27]. It has enhanced functionality and works on mobile as well as PC platforms. IBPI was accessible by PC/laptop in the feasibility study but recent ONS data highlights that 89% of internet users access the internet ‘on the go’ through mobile phones [28]. The website also contains improved co-produced content on both BD self-management and parenting, including two additional modules on managing parents’ and children’s sleep and anxiety. This will be compared with treatment as usual as there is no specific alternative support that parents with bipolar are systematically offered in the NHS.

## Objectives

### Hypothesis

IBPI plus treatment as usual (IBPI + TAU) will be superior to treatment as usual (TAU) at 24 weeks in improving child emotional and behavioural outcomes and will be cost effective.

### Primary objective

1. To determine the clinical effectiveness of IBPI on child behavioural and emotional problems at 24 weeks, measured using the Strengths and Difficulties Questionnaire (SDQ [29]).

### Secondary objectives

1. To determine the clinical effectiveness of IBPI on the secondary outcomes:

i) Child behavioural and emotional problems at 48 weeks, measured using the SDQ.
ii) Parenting competence, confidence, and stress at 24 and 48 weeks, measured using the Parenting Scale (PS [30]), the Parenting Sense of Competency Scale (PSOCS [31]), and the Parenting Stress Index Short Form (PSI-4-SF [32]).
iii) Parental mood (self-rated mania and depression) at 24 and 48 weeks, measured using the Internal States Scale (ISS [33]), the Centre for Epidemiologic Studies Depression Scale (CES-D [34, 35]), the Altman Self Rating Mania Scale (ASRM [36], the Generalized Anxiety Disorder Scale (GAD-7 [37]), and the Life Chart Method – Retrospective (LCM-r, adapted from the NIMH-LIFE [38]).
iv) Family coherence at 24 and 48 weeks, measured using the Confusion, Hubbub, and Order Scale (CHAOS [39]).

2. To determine the cost effectiveness of IBPI:

i) The primary economic objective is to compare the cost-utility of IBPI + TAU vs. TAU assessed at 24 weeks with effects measured in terms of quality-adjusted life years (QALYs) generated from the Child Health Utility 9 Dimension measure of health-related quality of life (CHU9D [40]).
ii) The secondary economic objectives include:

- Cost-utility analysis using QALYs at 48 weeks.
- Cost-utility analysis using QALYs at 24 and 48 weeks including costs and effects for the parent in addition to the child, with QALYs for the parent measured using the EQ-5D-3 L measure of health-related quality of life [41].
- Cost-effectiveness analysis using the primary clinical outcome measure (SDQ) at 24 and 48 weeks.

3. Obtain views of IBPI recipients on their experiences of IBPI:

i) A feedback survey will be sent to participants in the IBPI + TAU arm after 24-weeks to determine their levels of intervention use, and, if applicable, their opinions of the website. Only participants who have consented to be contacted for the qualitative study will be sent the survey.
ii) Qualitative interviews will be conducted with a sub-sample of participants who have completed the feedback survey. The sub-sample will be based on (i) stratification variables and (ii) levels of intervention use (determined by survey responses). The topic guide for these interviews will include questions about participants’ perceptions of what has changed following IBPI, the factors which influenced their level of engagement, and their recommendations for improvement.

### Patient and public involvement (PPI)

The PPI lead for the project has lived experience of bipolar and psychosis symptoms and is a grant holder for the study. They chair the service user reference group (SURG), which consists of individuals with lived experience of BD and parenting. SURG meetings will take place throughout the trial, overseeing all aspects of the work from intervention update and recruitment planning to development of study materials and plans for implementation and dissemination. The PPI lead also coordinated PPI input into the co-production of the updated IBPI intervention and to the refinement of data collection captured via the Research Electronic Data Capture (REDCap) system (a secure online database for collecting and storing research data). The Trial Steering Committee (TSC) includes representation from individuals with lived experience of BD and parenting.

### Trial design

**Fig. 1:**
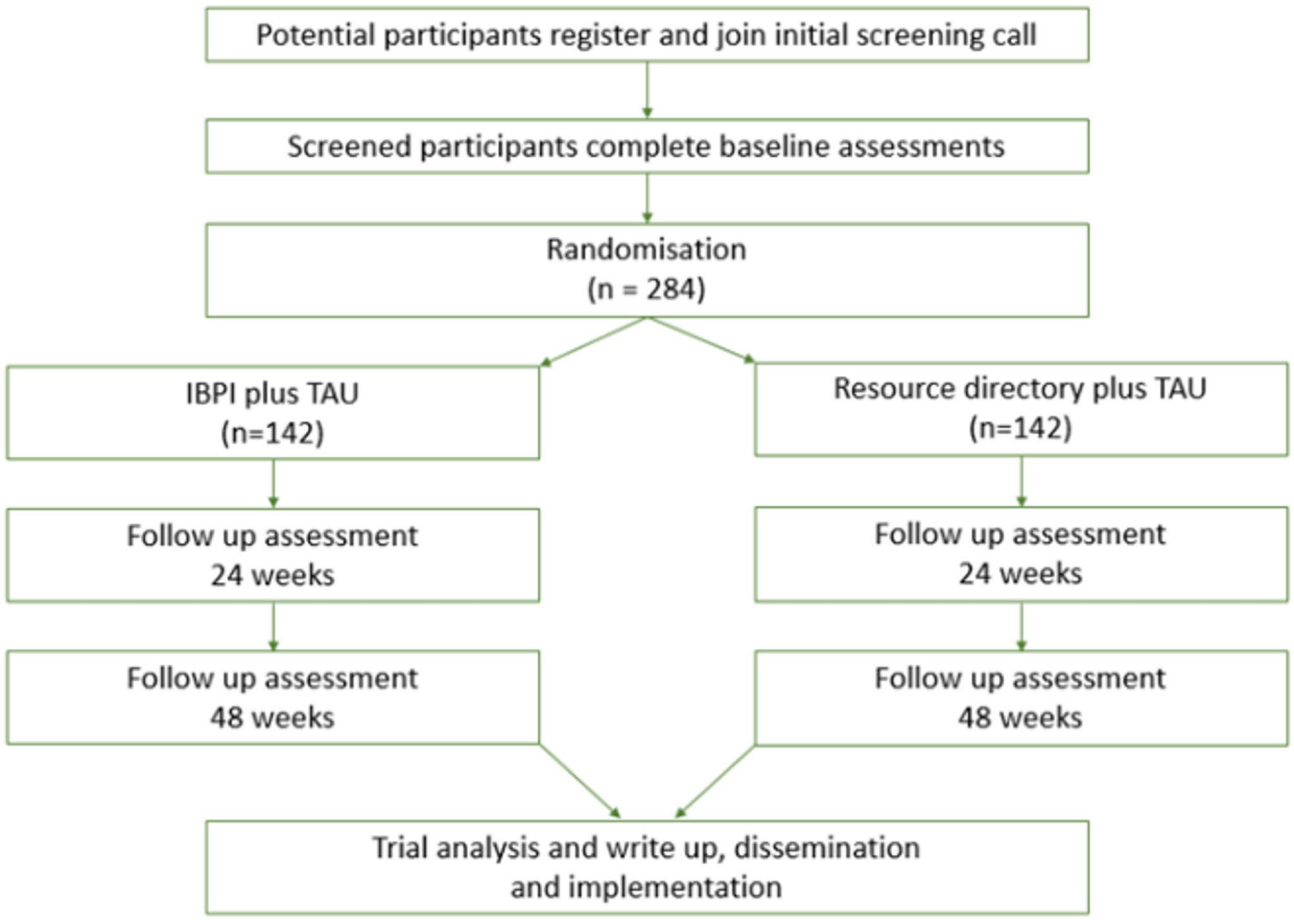
Participant flow through the study

This is a UK-based online-randomised controlled effectiveness and cost-effectiveness trial with nested qualitative study. Participants are allocated at a 1:1 ratio to TAU or IBPI + TAU. Stratification variables for randomisation are (1) number of previous bipolar episodes (3 levels; 1–7, 8–19, or > = 20), and (2) and whether or not their partner is receiving mental health care (3 levels; yes, no, or n/a – no partner).The trial design has been informed by the Medical Research Council [42] and SPIRIT [43] guidelines. Trial oversight is provided by a TSC and an Independent Data Monitoring and Ethics Committee (DMEC). See Fig. 1 for details of the participant’s journey through the trial.

## Methods: participants, interventions, and outcomes

### Study setting

This is a national trial across the UK including NHS patients referred through secondary mental health services as well as primary care referrals and opportunities for self-referral through social media campaigns.

### Eligibility criteria

Inclusion criteria:

- **Parent BD diagnosis**,** confirmed by structured clinical interview** [44]. This intervention is specifically tailored for individuals living with confirmed BD, so it is crucial this is established.
- **Index child aged 4–11 years with ≥ 10 h of face-to-face contact weekly.** The focus of this definitive trial is to support parents with BD of young children they are in regular contact with. This age group offers the opportunity for early intervention in a high-risk group likely to develop additional significant mental health issues in adolescence without appropriate support. We recognise families will often have several children, so the parent will identify an index child at baseline and will answer questions on that child for the duration of the trial. A reminder will be included in the 24 and 48-week surveys of which child was chosen. This reminder will be a piece of information that identifies the child to the parent but not to the trial team, e.g. the child’s favourite film.
- **Internet access.** This is required to ensure that people can access the online assessments and intervention. A limited number of internet dongles can be offered to participants without reliable internet connection.
- **Ability to provide informed consent.**
- **Resident in the UK.** The intervention has been designed for people in a UK context, including UK information on sources of information and support.

Exclusion criteria:

- **Parents with primary diagnosis of alcohol/other substance misuse.** Parents with primary substance use issues are likely to require different support to those for whom BD is the primary issue so the planned intervention would be less relevant.
- **Parents already receiving a parenting intervention and/or intensive psychotherapy.** The receipt of different forms of intensive psychological support at the same time could be confusing for the parent and would make it difficult to determine the impact of IBPI.
- **Index child in receipt of current psychological therapy.** There is a risk that messages from therapy could be different from what a parent is doing based on IBPI. It would also risk masking effects of the current intervention. Non-index children (other children aged 4–11 who they have more than 10 h of contact with a week), however, can currently be receiving psychological therapy.
- **Any child in the household within 4–11 age range identified by social services/multi-agency partners** due to current or ongoing child protection concerns.
- **Three cancelled or missed eligibility check calls** without providing at least 1 days’ notice.

An anticipated challenge to delivering a digital trial is false sign-ups through bots, to obtain payment. The team have put mechanisms in place to identify false sign-ups. Participants are also required to engage with a two-hour eligibility interview to determine whether they are eligible to participate. The trial team deem this as a substantial time commitment which will likely deter further engagement by those not actively wanting to engage with the full trial.

### Interventions

#### IBPI intervention

IBPI is an online resource for enhancing parenting skills and confidence as well as self-management in parents with BD. The IBPI program theory integrates mid-range cognitive social learning [45] and cognitive behavioural theories [46], and qualitative feedback from parents in the pilot study [23]. It has recently been argued that an affect–integration–motivation and attention–context–translation (AIM-ACT) framework may be helpful in understanding engagement with digital interventions and to provide recommendations to enhance engagement [47]. Consistent with the recommendations of this approach, IBPI was designed with hopeful positive content to promote positive affect. The intervention content is framed in ways that are self-relevant to participants and focuses on topics of high personal value, as informed by our co-production PPI work. It has also been designed to be used flexibly across different internet devices, again consistent with this framework.

IBPI aims to improve outcomes in the following ways, by:

1. Providing a normalising explanation of parenting and mood experiences, fostering engagement, reducing isolation, and challenging beliefs that stem from perceived unchangeable personal impairments.
2. Establishing a working model of the connections between mood, parenting, and child behaviour, which offers a rationale for adopting behaviour change strategies.
3. Offering specific positive parenting strategies within a framework to empower participants in experimenting with new parenting approaches and mood management.

The IBPI website has nine information modules each of which contains information and advice for parents relating to the topic as well as interactive and multimedia features including video clips, exercises, and self-evaluation activities. The site is aimed at normalising people’s experiences by providing lived experience examples of parenting with BD. It also aims to support parents to self-regulate and to challenge self-stigma. Data from our feasibility trial suggested that each module will take approximately 30 min to complete, with participants typically completing one module a week. The IBPI website will be accessible to participants in the IBPI arm of the trial 24 h a day, 7 days a week, from desktops, laptops, mobile phones, and tablets.

#### IBPI update

The IBPI platform update was codesigned during the first nine months of the study [27]. Changes made were based on the feedback from the feasibility study, input from academics and clinicians, and input from people with lived experience of parenting with BD. Lived experience feedback was provided during nine monthly sessions.

The modules contain accessible material including text and video clips. There are reflection exercises to support ongoing learning, videos providing a lived experience perspective, top tips and external resource links. Each module contains constructive and non-stigmatising content. All content considers the positive and negative impact bipolar can have on parenting, integrated with information on helpful parenting principles for each issue covered. The order of the modules is indicated on the website homepage, but participants are free to complete the modules in a different order if they choose to do so.

The titles for each module are as follows:

- Module 1: Parenting and bipolar disorder overview.
- Module 2: Benefits and challenges of bipolar in relation to parenting.
- Module 3: Understanding mood variation to help manage your child’s behaviour consistently.
- Module 4: Monitoring your mood.
- Module 5: Perfectionism, impulsivity and supporting your child to learn new skills.
- Module 6: Managing relationships and change.
- Module 7: Dealing with anxiety.
- Module 8: Managing sleep.
- Module 9: The importance of making time for yourself and planning ahead.

#### Control intervention

Individuals in the TAU group have access to a web page providing general information on sources of support for BD and parenting but no additional material. All participants in the control arm have the option of accessing the IBPI intervention at the end of the trial, if the intervention is confirmed safe based on the experience of those in the treatment arm.

### Outcomes

Prior to completing baseline measures, all participants complete the Structured Clinical Interview for DSM-5 [44] eligibility check to confirm BD diagnostic status and to provide clinical and sociodemographic data. This is supplemented by questionnaires at baseline, including a Sociodemographic Questionnaire specifically designed for this study and a Family Questionnaire providing additional details on both parents and the index child to characterise the sample. The inclusion of this self-report strategy has been informed by its successful implementation during our feasibility trial. Information collected includes:

- Parent’s age, marital status, gender, and ethnicity.
- Child’s age, gender and relationship to parent.
- Number of children per family.
- Whether the participant’s partner is in receipt of current mental health treatment.
- Number of previous BD episodes and hospitalisations.
- Parent current mental health treatment.
- Parent and partner education.
- Parent and partner work status.

### Primary outcome measure

To assess the child’s behavioural and emotional wellbeing, the SDQ will be completed by the parent for the index child. If the participant has multiple eligible children, they will select one child as their index child for their primary SDQ.

The SDQ has an established factor structure with strong internal consistency and test-retest reliability. In line with the aims of the study, high SDQ scores are consistently found to be strongly predictive of psychiatric disorders [29]. The SDQ is widely used and sensitive to change in parent and teacher mediated intervention studies, and in interventions for children of parents with serious mental illness (including [20, 48–51]. This was confirmed in our proof of principle and feasibility studies [23, 25].

The primary outcome is the SDQ total difficulties score for the index child at 24 weeks. This assessment point was chosen to allow sufficient time for participants to learn, adopt and implement behaviour changes to improve child wellbeing consistent with underpinning theory [45, 46, 52]. This is informed by feasibility data indicating: (i) over 95% of participants completed using IBPI by 3–4 months, leaving 2–3 months for this learning to be translated to child outcomes; (ii) SDQ slopes diverge baseline to 24 weeks between arms then plateau to 48 weeks (indicating maintenance in the second 24 weeks period). This primary outcome mirrors that of previous parenting intervention trials, aiding comparison of effects [25, 53].

### Secondary outcome measures

The SDQ can also be completed by parents about any other eligible children they have (i.e. aged 4–11 with whom they spend ≥ 10 h a week), to assess non-index children’s behavioural and emotional wellbeing, as well as to inform sensitivity analyses.

#### Parenting outcomes

PSOCS, PS and PSI-4-SF will be used to capture the multifaceted nature of parenting across confidence, competence, and stress. They all have strong psychometric properties and were sensitive to change in the feasibility study [23, 30–32, 54].

#### Parental mood outcomes

These will be measured with ISS, CES-D, ASRM, GAD-7, LCM. The LCM has been jointly adapted by the research team and clinical experts as a diary to help participants identify whether they have experienced episodes of mania, hypomania or depression during each follow up period by rating perceived severity of their mood experiences every 4 weeks. In incidences where participants have not diarised their moods, they are asked to provide reasons for not doing.

All these measures have evidence for validity, reliability and sensitivity to change [33, 34, 36–38].

#### Family functioning

Family functioning will be measured with the CHAOS-9, as a reliable, sensitive measure correlated with a wide range of physical, emotional, and academic outcomes in children [39, 55].

The selection of these outcome measures was informed by Retzer et al.’s recent Core Outcome Set for use in community-based bipolar trials qualitative study [56]. Specifically, it identified domains of measurement critical to community-based BD trials, of which the present study’s measures cover the core domains of connectedness, BD symptoms, wellbeing, and quality of life.

#### Economic measures

Data on services used by the child and the parent to estimate costs will be collected from parents using adapted versions: (i) the Child and Adolescent Service Use Schedule (CA-SUS) covering all health education or social care services used by the index child and (ii) the Carer Service Use Schedule (CARER-SUS) which covers all health services used by the parent plus productivity losses (time off work due to own health or child’s health and support needs). These measures were designed for application to populations with mental health difficulties and have been successfully employed in multiple studies (for example [57, 58]),. Both measures will be completed at baseline (covering the previous 3 months) and at the 24 and 48-week follow-up points (covering the period since last interview). Data on IBPI use will be collected separately by the research team.

Data on health-related quality of life, using measures capable of generating QALYs, will be collected using the CHU9D measure for the child [59, 60] and the EQ-5D-3 L measure for the parent [61]. The CHU9D consists of 9 questions (covering worry, sadness, pain, tiredness, annoyance, schoolwork/homework, sleep, daily routine and ability to join in activities), each with 5-level responses. The measure is designed for self-completion by the child, with guidance for proxy completion for younger children (those under the age of 7). For this trial, the measure will be completed by the parents on behalf of their children. The EQ-5D-3 L measure consists of 5 questions (covering mobility, self-care, usual activities, pain/discomfort and anxiety/depression), each with 3-level responses. Both measures will be completed at baseline, 24 and 48-week follow-up points. Utility values for each health state at each time point will be estimated using the UK adult general populations’ preference weights for the CHU9D [40] and the EQ-5D-3 L [62]. QALYs will be estimated for the defined period using a linear interpolation to calculate the area under the QALY curve [63].

#### Feedback survey

To supplement the feedback interviews, all participants in the intervention arm who have consented to be contacted for the qualitative aspect of the trial will be sent a feedback survey. The survey will combine multiple choice questions with open questions where participants can provide free text responses. The feedback survey will establish participants’ level of intervention use and, if relevant, will ask participants their opinion of the intervention, and whether they experienced any changes from using the intervention.

#### Feedback interviews

A subset of participants (*n* = 15–20) from the IBPI + TAU arm who have completed the feedback survey, will be selected with maximum variance sampling on (i) stratification factors and (ii) levels of intervention use (determined by responses to the feedback survey) to participate in a feedback interview. The purpose of this interview will be to gain a more in-depth understanding of participant experiences with the IBPI website, such as what worked well, what still needs to be improved, and why. The feedback interview will also explore participants’ appraisal of it, what they have learned from the intervention, and patterns of website use. Importantly, this interview will also ask participants to share their perceptions of what has changed for them and their child because of their completion of the intervention and will look to identify any barriers/facilitators to engagement. The topic guide for these interviews will be co-developed with our SURG.

### Participant timeline

See Fig. 1 for participant flow through the study. After registration of interest participants will be provided with the PIS and consent form by the research team. On receipt of completed informed consent form, the participant will be sent an invitation for a diagnostic eligibility assessment.

Clinical and qualitative interviews will be conducted using the live video facility of MS Teams or phone depending on participant preference. All self-report measures will be completed online using REDCap. Demographic assessments will be collected at baseline only. Assessment of all outcome measures will be conducted at baseline, then again at both 24 and 48-weeks post randomisation. See Table 1 for a full schedule of assessments during the trial period.

**Table 1:**
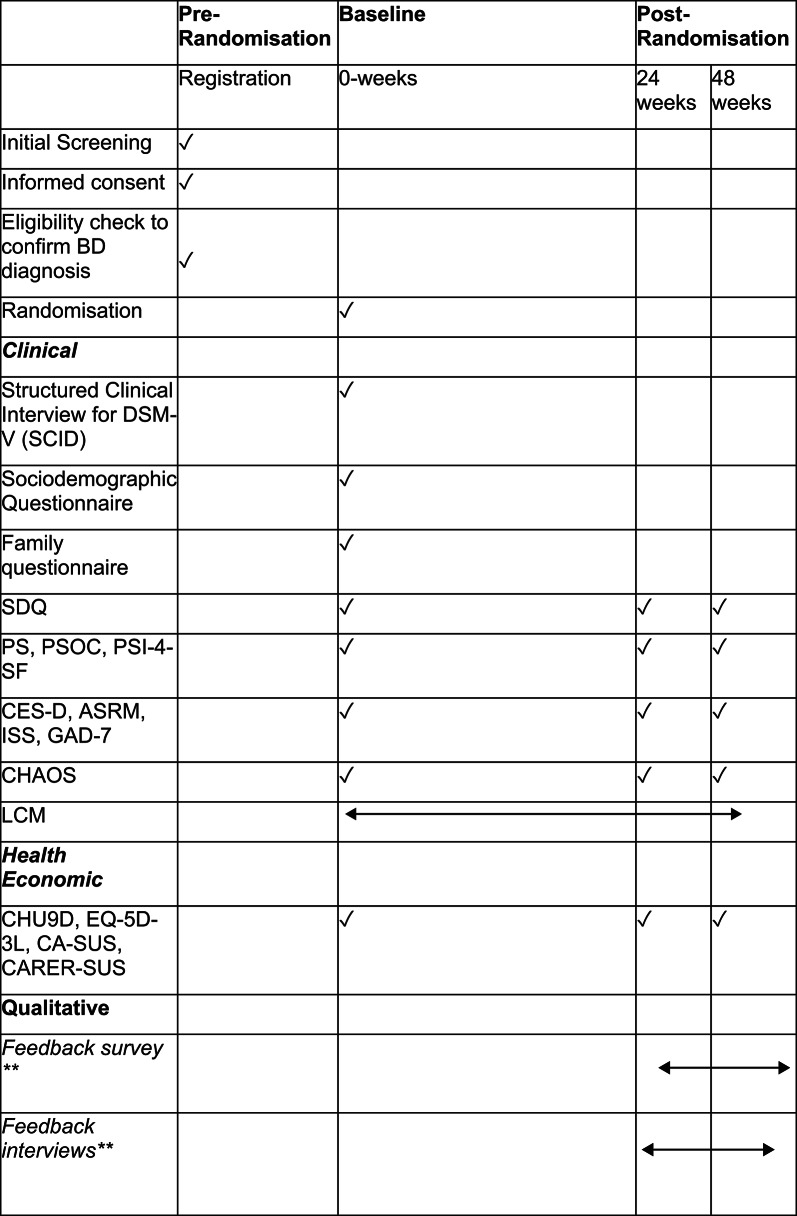
Schedule of assessments

*DSM-V (SCID)* Diagnostic and Statistical Manual of Mental Disorders, Fifth Edition, *SDQ* Strengths and Difficulties Questionnaire, *PS* Parenting Scale, *PSOC* Parenting Sense of Competency; *PSI-4-SF* Parent Stress Index 4 Short Form *CES-D* The Center for Epidemiological Studies-Depression, *ASRM* Altman Self-Rating Mania Scale, *ISS* Internal States Scale, *GAD-7* Generalized Anxiety Disorder Assessment, *CHAOS* Confusion, Hubbub and Order Scale, *LCM* Life Chart Method, *CHU9D* Child Health Utility 9-dimensions, *EQ-5D-3 L* EuroQol measure of health-related quality of life, *CA-SUS* Child and Adolescent Service Use Schedule, *CARER-SUS* Carer Service Use Schedule

**Feedback survey and interviews expected not to begin until after completion of the internal pilot

### Sample size

The trial is powered to detect a 2-point difference on the SDQ at 24 weeks with 90% power, using an analysis of covariance [ANCOVA] with a 5% significance level. In our pilot trial [23], a reduction of 2-points in the SDQ would have led to a 9% reduction in children scoring in the clinical range at follow-up. In line with Ford et al. this effect size would also be expected to reduce the odds of a child having a psychiatric disorder diagnosis within 3 years by 40–50% for each 2-point decrease in SDQ [48], which would be a clinically important reduction. PPI consultation also confirmed that this effect would be experienced as important given the simple and inexpensive nature of the intervention. The pooled standard deviation of the 24-week SDQ from the 76 children with baseline and 6-month outcome from our pilot trial was 6.46 [23].

Hence, to detect a difference of 2 points on the SDQ at 24-weeks, assuming a standard deviation of 6.46, and a correlation of 0.65 between baseline and week 24 [23, 64] a sample size of 256 participants is required for 90% power and 5% significance level. In practice, power will be increased slightly due to the use of constrained longitudinal data analysis.

The original sample size was agreed at 342. This assumed that there would be 75% retention of participants to the primary outcome follow-up time-point. During the study it was observed that the retention rate was much higher, at 90%. Given slower-than-anticipated recruitment into the study and assuming this higher rate of retention, the target sample size was reduced to 284 after approval from the funder on 21/02/2025, with all other assumptions remaining the same. The total sample size will be 284.

### Recruitment

The trial will recruit participants in two ways: through clinician referrals and self-referral. We will collect information on recruitment regularly to review the success of different approaches and to tailor recruitment to be as inclusive as possible. We will work with our lead Trust (Lancashire and South Cumbria NHS Foundation Trust), Bipolar UK and our SURG to maximise recruitment strategies.

#### Clinician referral pathway

The trial will recruit NHS patients from across the UK through secondary care mental health Trusts and GP practices. Recruitment will involve, clinical staff and clinical studies officers reviewing clinical caseloads and conducting medical record database searches to identify potential participants. Potential participants will be sent a letter or SMS text message that will introduce the study and signpost them to the study’s website (www.lancs.ac.uk/spectrum/ibpi) where they can register their interest. Researchers, clinical studies officers and clinical staff will also promote the study through relevant clinical teams and their service’s social media pages.

#### Self-referral pathway

Recruitment will be supported by targeted social media campaigns calling for self-referrals through Facebook, Instagram and Google ads. Similarly, trial partner Bipolar UK will also share recruitment information on their platforms to request expressions of interest. Additionally, the Research Delivery Network (RDN) and local researchers will work to advertise the study in both NHS and community settings, as well as host community outreach events.

The success of both referral routes will be regularly reviewed. The social media campaign will be adapted based on feedback from potential participants on how they became aware of the research.

Recruitment into the trial is ongoing and due to finish on 30th November 2025.

In our feasibility study [23], we were successful in recruiting to target and our participants were similar in profile to participants in face-to-face trials. However, the ethnic diversity of the group was low with over 90% of participants identifying as white British. To improve this, our PPI plan includes targeting people from minority ethnic backgrounds to ensure that recruitment and intervention materials are inclusive, and the recruitment strategy includes NHS and third sector providers that specifically support people from minority ethnic backgrounds. Our approach is guided by NIHR’s equality diversity and inclusion policy and informed by NIHR’s *Toolkit for: Increasing participation of Black*,* Asian and Minority Ethnic (BAME) groups in Health and Social Care Research* [65].

## Methods: assignment of interventions

### Randomisation

After consenting and completing baseline measures, participants will be randomised to either IBPI + TAU or TAU alone. Randomisation will be conducted using an online system (within the REDCap electronic data capture system) set up by York Trials Unit (YTU). The randomisation sequence has been generated by an independent statistician, not involved with the analysis of the trial. Its algorithm uses stratified block randomisation: stratification is based on the number of previous bipolar episodes (3 levels; 1–7, 8–19, or > = 20), and whether or not their partner is receiving mental health care (3 levels; yes, no, or n/a – no partner).

### Blinding

Participants will not be blinded to their intervention and will self-complete all outcome measures. Researchers involved in supporting follow-up assessments will be blind to treatment allocation as will the Chief Investigator, except where there is immediate risk of harm to a participant and this needs to be broken for safety reasons. The senior health economist will be blinded to trial allocation for analysis. Trial statisticians and the trial health economist analysing the data will not be blinded. The Trial Manager, and Trial Support Officer at YTU can view the group allocation but are not party to the unblinded results. Group allocation will be communicated via email (through the REDCap system) to the randomisation inbox to which only the Trial Manager and Project Administrator will have access. This is to monitor adherence to the algorithm for randomisation and allow for a mechanism of informing participants of their group allocation. Any queries regarding the intervention can then be discussed with the Trial Manager to ensure blind breaks do not occur with other blinded researchers. Participants will be reminded not to unblind researchers at each assessment point. Should an unblinding occur, subsequent assessment support for that participant will be conducted by an alternative researcher. All blind breaks will be reported to the DMEC and the TSC.

## Methods: data collection, management, and analysis

### Data collection and methods

Potential participants will register their interest via Microsoft Forms, which will ask for their consent to contact them about the trial and their contact details (phone number, email address, and postal address). This information is captured to allow a member of the research team to make direct contact with participants to discuss participation in the trial, answer questions, arrange the eligibility confirmation interview, contact about follow up assessments for those who are eligible, and share the results of the trial once it is complete.

### Optimising retention

Attrition is a key challenge faced by online trials [66]. Our retention strategy has been informed by previous studies run by Lancaster University, our team [23, 67], and a recent meta-analysis [68]. To maximise retention in the current study we will:

- Include an explanation in the Participant Information Sheet describing why data completion at follow-up is important.
- Only randomise participants once they have completed the measures at baseline.
- Send participants scheduled email and telephone reminders to prompt engagement with the intervention and with each assessment point, based on previously successful strategies [69]. If participants still do not complete measures, we will send the measures in the post to be completed by pen and paper.
- Pay participants for completing questionnaires at each assessment point: £40 for completing the SCID and baseline measures, £10 for completing 24-week measures, £10 for completing 48-week measures, £5 for completing the feedback survey and £40 for those who attend a feedback interview. There is some evidence to suggest that paying participants improves retention [65, 70].
- Allow participants who may be unable to complete all follow-up measures to only complete some of them, with an emphasis on the primary outcome measure (SDQ).
- Allow participants to complete assessments at times and locations of their choosing by using online self-report measures.
- We will review attrition by key characteristics throughout the trial to identify patterns and bring in strategies that might support specific groups to continue to take part.

### Internal pilot

The feasibility work demonstrated that IBPI is acceptable, safe, and potentially helpful to children and parents [23]. However, for the current study an internal pilot was included to confirm recruitment to scale in a national definitive trial (see Table 2). The internal pilot recruitment target was based on the original sample of 342 participants within a 24-month recruitment window. For an 8-month internal pilot, (35% of the total window for recruitment) the target was *n* = 75 (22% of total study target, beginning *n* = 1 per month in month 1, to *n* = 11 per month in month 5 rising to *n* = 16 in month 7 and *n* = 17 per month in month 8). This allowed for staggered set-up of Patient Identification Centres (PICs) and time to fully optimise online recruitment approaches. Ongoing recruitment figures will be regularly reviewed at trial management meetings and the TSC.

**Table 2:** STOP/REVIEW/GO criteria for the internal pilot

	Red - STOP	Amber - REVIEW	Green – GO
Total number of participants recruited after completion of the internal pilot	0–44 participants (< 60% of recruitment target)	45–74 participants (60–99% of recruitment target)	>=75 participants (> = 100% of recruitment target)
Actions	Stop – unless demonstratable mitigating circumstances and strategies to mitigate and improve recruitment	Discuss with TMG and TSC strategies to improve recruitment, including additional sites and proceed with funder permission	Proceed

### Data management

The results of the eligibility interviews are recorded by the researcher and provided to YTU (by inputting to REDCap) for randomisation of those who are eligible. All other patient reported outcomes (PROMs) are collected directly from participants using REDCap. This system was extensively tested by the research team and PPI members to ensure accuracy at launch with the start of recruitment.

We are seeking to minimise missing data whilst also maintaining the acceptability of the assessment procedure for participants. There are a number of compulsory questionnaires namely SDQ, CHU9D, EQ-5D-3 L (primary clinical and health economic outcomes), CES-D, ASRM, GAD-7 (to monitor for mood issues during the trial and offer support if needed), and covariates required for the randomisation procedure. Data from feedback surveys will be collected directly from participants using Lancaster University hosted Qualtrics. Qualitative interviews will be recorded using encrypted recording software and then transcribed and saved in de-identified form. Any identifiable information will be stored separately. All study data will be securely stored in line with ethical approval on password protected NHS and University systems. YTU will host the anonymised data for the monitoring of data and data analysis at the end of the trial.

### Statistical methods

#### Quantitative data analysis

Analysis will be undertaken on an intention-to-treat basis, using two-sided tests, and a 5% significance level. Full details of the analysis will be included in a Statistical Analysis Plan (SAP) which will be developed by the Trial Statistician prior to analysis and approved by the Trial Management Group (TMG) and TSC.

#### Primary outcome analysis

To make use of all available observations from all-time points in the study, the estimate for the 24 week between-groups difference in SDQ total score will be derived from a constrained longitudinal data analysis (cLDA) model [71]. This model will allow a participant to be included in the analysis if they have provided the primary outcome at any of the post-randomisation timepoints, minimising the number of participants excluded from the model. The model will be a linear mixed-effects model, featuring SDQ total score for the index child as the outcome, and will include allocation group, time-point, and stratification factors as fixed effects, and participant identifier as a random effect. Intervention group-by-time-point interaction effects will be included for each of the 24-week and 48-week time-points, thereby making no assumptions about the shape of the SDQ score trajectory over time. The model will be constrained so that the expected baseline SDQ scores are equal in the two groups [71]. Parameter estimation will use maximum likelihood, with an unstructured covariance matrix. The between-groups difference in SDQ score at 24-weeks (primary outcome) and 48-weeks (secondary outcome) will be extracted (i.e. the respective group-by-time-point interaction effect estimates) and reported from this model. No missing data imputation will be used in the primary analysis – but the outcomes method to handle missing responses will be used when scoring the SDQ, as detailed in the SAP.

#### Secondary outcome analysis

Secondary outcomes will also be analysed using cLDA models, with adjustment for the stratification factors.

A planned subgroup analysis will be performed on the primary outcome variable and include investigation of any differential impact of the intervention in the presence of partner receiving mental health support, the number of children within the household and, index child gender.

Feedback survey data will be summarised using descriptive statistics, reporting frequencies of responses to get a broad sense of how usable the website was for participants.

#### Qualitative data analysis

Content analysis will identify the key points being made in the open questions of the feedback survey. Based on previous experience, it is anticipated that most of the responses to the open questions will be fairly brief and telegraphic.

Analysis of the anonymised interview transcripts will follow the framework approach of Ritchie and Spencer [72]. The initial framework will be based on the need to understand what people felt changed as a result of engaging with IBPI as well as patterns of use and what influenced these. Specifically, we will ask about engagement with the IBPI intervention, what (if anything) they are doing differently, due to their use of IBPI; explore their rationale for making these changes; and elucidate the processes by which they felt able to make these. This initial framework will evolve through familiarisation and indexing to produce final themes. We will interview approximately 15–20 participants using topic guided interviews, sampled across stratification variables and levels of website use.

Findings from the survey and interviews will be triangulated using narrative synthesis [73, 74]. This will be developed through stakeholder workshops with the PPI group in which the summary data will be presented, and the group will be invited to question and interpret the data.

We have taken a primarily inductive approach to allow participants to generate their own theories of change. This ensures that we understand what has changed from their perspective and avoids suggesting any mechanisms that may not be valid. Using purposive sampling across our stratification variables we aim to understand the full range of participant experiences, and how these vary across different contexts. We will then interpret these findings in light of our programme theory and develop this accordingly. We believe that ongoing iterative development of this theory is crucial to understand how best to optimise the intervention as it is rolled out in practice. The qualitative interviews will also explore implementation issues which are crucial to facilitate effective delivery following the trial.

#### Economic analysis

In line with the clinical analyses, economic analyses will be on an intention-to-treat basis, and full details will be included in a Health Economic Analysis Plan (HEAP) which will be developed by the Trial Health Economist prior to analysis and approved by the TMG and TSC.

The primary economic analysis is a cost-utility analysis at 24-weeks with effects measured in terms of QALYs from the CHU9D and taking the NHS/Personal Social Services perspective preferred by NICE. Secondary economic analyses include: (a) primary economic analysis repeated at 48 weeks; (b) cost-effectiveness analysis using the primary clinical outcome measure (SDQ) at 24 and 48 weeks and taking the NHS/Personal Social Services perspective; and (c) cost-utility analysis focused on combined child and parent costs and QALYs at 24 and 48 weeks and taking a broader perspective, including parental productivity losses. Sensitivity analysis will explore the impact of excluding influential cost outliers (those in the 99th cost percentile [75]).

Costs will be estimated using appropriate UK unit costs for the most recent financial year at the time of analysis, including NHS reference costs for hospital resource use [76] and national unit costs of health and social care services for community-based resource use [77]. Replacement cost approach will be used for estimating the costs of productivity losses [78]. The cost of the intervention will focus on the maintenance costs of the IBPI application, including server maintenance, software updates, and technical support [79]. Application development costs will be excluded on the grounds that they are sunk costs.

Resource use will be reported descriptively as mean (standard deviation) and percentage of participants using the resource item. Mean difference in total cost and QALYs per participant between the randomised arms will be estimated using bootstrapped generalised linear models (GLM), adjusted for covariates in line with the clinical analyses, plus the baseline variable of interest (baseline cost, utility score, SDQ score).

Cost-effectiveness will be assessed through the calculation of incremental cost-effectiveness ratios (ICERs; the additional cost of one intervention compared with another divided by the additional effects) for any cost–outcome combinations involving a trade-off between costs and effects such that one group generates higher costs and greater benefits compared with the other (lower costs and higher outcome combinations are considered ‘dominant’). Uncertainty will be explored using cost-effectiveness planes and cost-effectiveness acceptability curves (CEACs; [80, 81]) based on the net-benefit approach [82, 83]. Cost-effectiveness planes plot the adjusted mean differences in total cost and effects calculated using the bootstrapped results associated with the regression models noted above. Cost-effectiveness acceptability curves will be derived by calculating the proportion of bootstrapped estimates that are cost-effective across a range of willingness-to-pay thresholds, to show the probability that the intervention is cost-effective across different threshold values.

#### Missing data

We are seeking to minimise missing data whilst also maintaining the acceptability of the assessment procedure for participants. There are a number of compulsory questionnaires namely SDQ, CHU9D, EQ-5D-3 L (primary clinical and health economic outcomes), CES-D, ASRM, GAD-7 (to monitor for mood issues during the trial and offer support if needed), and sociodemographic questions relevant to the randomisation procedure. Where missing item data exists, the scoring manual will be consulted to determine the score. Mean imputation will be used when scoring manuals do not provide guidance on how to deal with missing item data. Mean imputation will occur only when ≤ 25% of the items are missing. The primary analysis model assumes that data will be missing at random (i.e. the probability of missing data may depend on the observed data and treatment group, but not on the unobserved responses). Where possible, the appropriateness of this assumption will be checked via observation, ensuring there is no obvious imbalance between the arms, i.e. no differential attrition. Appropriate sensitivity analysis will be performed, with full details provided in the SAP.

## Methods: monitoring

### Data monitoring

There will be monthly meetings of the TMG (including statisticians, health economists, qualitative, clinical and lived experience expert grant holders) to ensure the successful delivery of the study. These will be supplemented with weekly operational meetings of research staff and the Principal Investigator to discuss specific issues with recruitment, retention, data collection and risk issues. Independent oversight is provided by a TSC, chaired by an expert in child clinical psychology and includes members with expertise in BD, people with lived experience of BD, and members with expertise in statistics and health economics. Each TSC meeting will be preceded by a meeting of the DMEC chaired by an experienced child and adult clinical psychologist with other DMEC expert members from health economics and statistics. The DMEC has a specific role to be able to review unblinded data and to make any necessary recommendations to the TSC on any safety or ethical issues that might emerge from evaluation of these data. Both TSC and DMEC will meet at least twice a year throughout the study.

### Adverse events

Adverse events for the study will be classified as low risk (evidence of high level of distress or concerns for possible risk of harm to individual or safeguarding risk) or high risk (clear evidence of immediate and serious risk to participant’s life or child welfare). Such events will be detected through participant interviews, direct contact (telephone or email) from participant to researchers or trial manager, or through red flag alerts on specific online assessment items. For low-risk events participants will be sent a supportive email signposting them to relevant third sector and NHS support options. For high-risk events the researcher will contact relevant services. In case of immediate risk to life the researcher will contact 999. For immediate and/or serious child welfare risk social services safeguarding teams will be contacted. All risk events will be reported and recorded. Events will be discussed with the supervising clinician. High risk events will be reviewed by TSC chair to evaluate possible relatedness. If related the CI will be unblinded and the sponsor, ethics committee and funder will be informed. Risk events will be reviewed by the DMEC for both trial arms.

### Ethics and auditing

Although no specific audits are planned, these can be requested by NIHR, TSC, DMEC and sponsor as well as other study partners.

## Ethics and dissemination

### Research ethics approval

This study has received NHS REC approval (West Midlands – Solihull Research Ethics Committee 22/WM/0200).

### Protocol amendments

Any protocol amendments will be reviewed by the Sponsor. These will also be shared with the Research Ethics Committee (REC) for approval prior to implementation.

### Consent

Participants must provide their consent to take part in the study and will be asked for their consent up to four occasions. Firstly, participants will register their interest to participate and consent to be contacted using MS Forms, which is accessible from the trial webpage www.lancaster.ac.uk/health-and-medicine/research/spectrum/research/ibpi/. The consent to contact form provides consent for their data to be stored in line with UK General Data Protection Regulation (GDPR) laws and the Data Protection Act 2018. Data includes phone number, email address, and postal address. Following an initial screening call if deemed potentially eligible, the researcher completing the screening call will send a link to the full trial consent form (on the cloud-hosted REDCap server). Completion of the full trial consent form provides both consent to participate in the full trial as well as consent to be invited to participate in the qualitative aspect of the trial for those randomised to the IBPI + TAU arm. Invites to feedback surveys will occur following a participant’s completion of their 24-week assessment, or if incomplete, four weeks after the assessment window has opened, and all prompts to complete the assessment have been sent. Participants will be sent a link via email to Qualtrics to complete the consent form. Once complete they will be directed to the feedback survey. A sample of participants who have completed their survey (up to *n* = 15–20) will be invited to a feedback interview via email. The email will contain a link to complete the feedback interview consent form which will be hosted on Qualtrics. Participants will not be invited to complete a feedback survey or feedback interview if they have not agreed to be contacted for the qualitative aspect of the trial, or if they have moved into the 48-week post randomisation assessment window.

### Confidentiality

Participant data will be shared with third parties (social services/clinical teams/emergency services) only when serious and/or immediate harm to themselves or others is likely, or a safeguarding concern is raised. Participants will consent to information being shared in this way when they complete the full trial consent form. Participants’ personal data will be stored on Lancaster Teams and on the secure cloud-hosted REDCap server, with access only to delegated trial team members. Baseline and follow-up assessments will be stored on the secure cloud-hosted REDCap server. Responses to the feedback survey will be stored on a secure Lancaster Qualtrics account with access only to delegated, unblinded trial team members.

### Dissemination

Findings from the study will be disseminated widely. The report of the trial outcome will be published in a peer reviewed journal article providing details in line with CONSORT guidelines [84]. The work will also be shared through lay articles for service user groups and through conference presentations. Authorship for all publication will be consistent with ICMJE guidance [85]. Findings will also be shared on the study website and study specific social media accounts. Study data will be stored at Lancaster University on trial completion. Access requests for the anonymised data will be considered by the TMG. Other trial materials are available on request from the research team.

## Discussion

Parents with BD want support with parenting but options that are both acceptable to them and effective are lacking. Such support has the potential to address immediate emotional and behavioural needs of children and may also mitigate risk of future complex mental health issues in these children. There are no definitive trials specifically offering support for parents with BD of young children. There has been a previous qualitative study of practitioner’s experiences of sharing parent self-help workbooks with parents with a range of mental health problems [22], including BD but not tailored specifically for parents with BD nor formally evaluated. The only feasibility studies are those conducted by our own team [23, 25]. Although systematic reviews indicate that online parenting interventions can improve child outcomes and parenting [21], none of these have been specifically designed for parents with BD.

This is the first definitive trial of a digital intervention integrating support for living well with BD and enhancing parenting. Feasibility work has shown this approach to be acceptable and feasible. This study will determine the effectiveness and cost effectiveness of the approach. The intervention has been co-produced with individuals with BD to ensure that the modules are accessible, engaging and straightforward to use for busy parents with very limited time. The trial will be delivered online to maximise access and to optimise cost efficiency of the trial itself. Although the feasibility study showed signals of benefit in terms of parenting and child outcomes, there were a number of weaknesses that this study will endeavour to address [23]. These changes have been informed by our qualitative work from the feasibility study, feedback from our SURG during that study, PPI consultation with parents with BD in preparation for this application, and expert guidance from clinical members of the applicant team. These changes will retain the simplicity and ease of navigation of the IBPI intervention with relatively brief modules combining accessible text information, video, interactive exercises and opportunities for self-reflection. These elements were valued by participants who often reported having little time between their parenting roles, their BD challenges and other day-to-day responsibilities. As indicated in the introduction, the needs of parents with BD are specific as they are living with the challenges of mood issues themselves, they have children with elevated rates of emotional and behavioural problems at risk for BD, and they are often reluctant to share their concerns with their clinical teams. This approach has therefore been designed specifically with these concerns in mind. In addition, the study takes a rigorous approach to determining inclusion criteria in particular parental diagnostic status. This is done using the gold standard DSM-V clinical interview, research edition (SCID-5-RV [44]). By adopting this approach, it is possible to ensure all inclusion criteria are fully met whilst also taking a flexible approach to recruitment pathways including clinical and self-referral. This flexibility is crucial to ensure that the sample reflects the reality that many people with BD are not in clinical mental health services consistently but are still living with significant challenges linked to mood and parenting.

A key problem with digital interventions is implementation. There are many studies that have demonstrated effectiveness in mental health but have not been implemented [86]. Similarly, there are many interventions that are available for which evidence is lacking. The IBPI intervention is designed to be simple and easy to implement. As it does not require direct clinician time in delivery or oversight it addresses concerns reported by NHS staff in recommending or hosting other digital interventions [87]. Consistent with contemporary research, we recognise that digital interventions need to evolve and therefore have specified both the theory of change for this approach as well as its fundamental characteristics. Future iterations of the intervention will therefore retain these key elements but permit evolution of functional elements of the platform. To maximise likelihood of successful implementation we are partnered with LSCFT, a digital pathfinder Trust, and with the national charity Bipolar UK, as well as liaising with NHS England.

If successful, the IBPI intervention has the potential to benefit parents with BD and their children including potentially reducing future risk of severe mental health challenges in the latter. It is also designed to be able to be implemented rapidly at scale and at low cost if effective. This approach is not intended to replace more intensive face-to-face support for parents such as crisis support. It is intended as a potentially helpful option to supplement other care. Future research is needed into tailored intensive parenting support for parents with BD.

## Data Availability

No datasets were generated or analysed during the current study.

## Abbreviations

ADHD: Attention Deficit Hyperactivity Disorder
ASRM: Altman Self-Rating Mania Scale
BD: Bipolar Disorder
CARER-SUS: Carer Service Use Schedule
CA-SUS: Child and Adolescent Service Use Schedule
CES-D: The Centre for Epidemiological Studies-Depression
CHAOS: Confusion, Hubbub and Order Scale
CHU9D: Child Health Utility Questionnaire
DMEC: Data Monitoring and Ethics Committee
DSM-5: Diagnostic and Statistical Manual of Mental Disorders, Fifth Edition
EQ-5D-3L: EuroQol measure of health-related quality of life
GAD-7: Generalized Anxiety Disorder Assessment
IBPI: Integrated Bipolar Parenting Intervention
IDMC: Independent Data Monitoring Committee
ICMJE: International Committee of Medical Journal Editors
ISS: Internal States Scale
LCM: Life Chart Measure
NICE: National Institute for Health and Care Excellence
NIHR: National Institute for Health Research
PPI: Public and Patient Involvement
PS: Parenting Scale
PSI-4-SF: Parent Stress Index 4 Short Form
PSOC: Parenting Sense of Competency
REDCap: Research Electronic Data Capture
SCID-5: Structured Clinical Interview for DSM-5
SDQ: Strengths and Difficulties Questionnaire
SURG: Service user reference group
TAU: Treatment as Usual
TMG: Trial Management Group
TSC: Trial Steering Committee
YTU: York Trials Unit
